# Rice actin binding protein RMD controls crown root angle in response to external phosphate

**DOI:** 10.1038/s41467-018-04710-x

**Published:** 2018-06-11

**Authors:** Guoqiang Huang, Wanqi Liang, Craig J. Sturrock, Bipin K. Pandey, Jitender Giri, Stefan Mairhofer, Daoyang Wang, Lukas Muller, Hexin Tan, Larry M. York, Jing Yang, Yu Song, Yu-Jin Kim, Yang Qiao, Jian Xu, Stefan Kepinski, Malcolm J. Bennett, Dabing Zhang

**Affiliations:** 1 Joint International Research Laboratory of Metabolic & Developmental Sciences, State Key Laboratory of Hybrid Rice, SJTU-University of Adelaide Joint Centre for Agriculture and Health, School of Life Sciences and Biotechnology, Shanghai Jiao Tong University, Shanghai, 200240 China; 2 Centre for Plant Integrative Biology, School of Biosciences, University of Nottingham, Loughborough Leicstershire, LE12 5RD Nottingham, UK; 3 National Institute of Plant Genome Research (NIPGR), New Delhi, 110067 India; 4 Department of Pharmaceutical Botany, School of Pharmacy, Second Military Medical University, Shanghai, 200433 China; 5 National Key Laboratory of Crop Genetic Improvement, Huazhong Agricultural University, Wuhan, 430070 China; 6 Department of Biological Sciences and Centre for BioImaging Sciences, National University of Singapore, Singapore, 117543 Singapore; 7 Centre for Plant Sciences, Faculty of Biological Sciences, University of Leeds, Leeds, LS2 9JT UK; 8 University of Adelaide-SJTU Joint Centre for Agriculture and Health, School of Agriculture, Food and Wine, University of Adelaide, Waite Campus, Urrbrae, 5064 SA Australia

## Abstract

Root angle has a major impact on acquisition of nutrients like phosphate that accumulate in topsoil and in many species; low phosphate induces shallower root growth as an adaptive response. Identifying genes and mechanisms controlling root angle is therefore of paramount importance to plant breeding. Here we show that the actin-binding protein Rice Morphology Determinant (RMD) controls root growth angle by linking actin filaments and gravity-sensing organelles termed statoliths. *RMD* is upregulated in response to low external phosphate and mutants lacking of RMD have steeper crown root growth angles that are unresponsive to phosphate levels. RMD protein localizes to the surface of statoliths, and *rmd* mutants exhibit faster gravitropic response owing to more rapid statoliths movement. We conclude that adaptive changes to root angle in response to external phosphate availability are RMD dependent, providing a potential target for breeders.

## Abstract (web summary)

The orientation of plant roots responds to gravity and influences nutrient acquisition. Here the authors show that the formin RMD buffers movement of specialized gravity-sensing organelles and report enhanced RMD expression during phosphate deficiency that could alter root angle to improve phosphate uptake.

## Introduction

Root architecture critically influences nutrient and water uptake efficiency in crops^1–3^. Rice root system contains primary roots, crown roots, and lateral roots^1^. The potential impact of improving nutrient use efficiency in crops through manipulating root architecture has been termed a “Second Green Revolution”^4^. Land plants have developed sophisticated mechanisms to forage for soil resources. For example, plant roots employing tropic responses to link their direction of growth with cues such as gravity^3,5,6^.

In many natural and agricultural ecosystems, soil phosphate is a major constraint for crop productivity due to its accumulation at the soil surface^7,8^. To adapt to low phosphate availability in soil and increase phosphate uptake efficiency, plants alter their root angle to increase phosphate acquisition at minimum cost^2,9,10^. The growth orientation of root branches is often actively maintained with respect to gravity, in which case the roots are said to have a gravitropic setpoint angle or GSA^11,12^. Recent work in Arabidopsis has shown that lateral roots with non-vertical GSAs are distinguished from the primary roots with ~vertical GSAs by the action of an auxin-dependent offset mechanism that counterbalances underlying gravitropic response in the root branch^11,13^. In this system, root growth angles are the product of the relative magnitude of the gravitropic and the counterbalancing offset components, with variation in either providing a means to alter GSA^10,11,13^. The molecular and genetic basis of the control of GSA in Arabidopsis is beginning to be elucidated^10,11,13–16^. In contrast, few genes and/or molecular mechanisms regulating root growth angle in crops have been identified to date^17^ and none have been linked to nutrient availability.

Here we report that the rice actin-binding protein RMD acts to dampen root gravitropism. Furthermore, we show that RMD expression is upregulated in response to phosphate and that phosphate-dependent changes in root angle are RMD dependent. We propose that RMD acts to fine-tune root angle in response to soil phosphorus availability.

## Results

### Actin-binding protein RMD dampens root gravitropsim

**Fig. 1:**
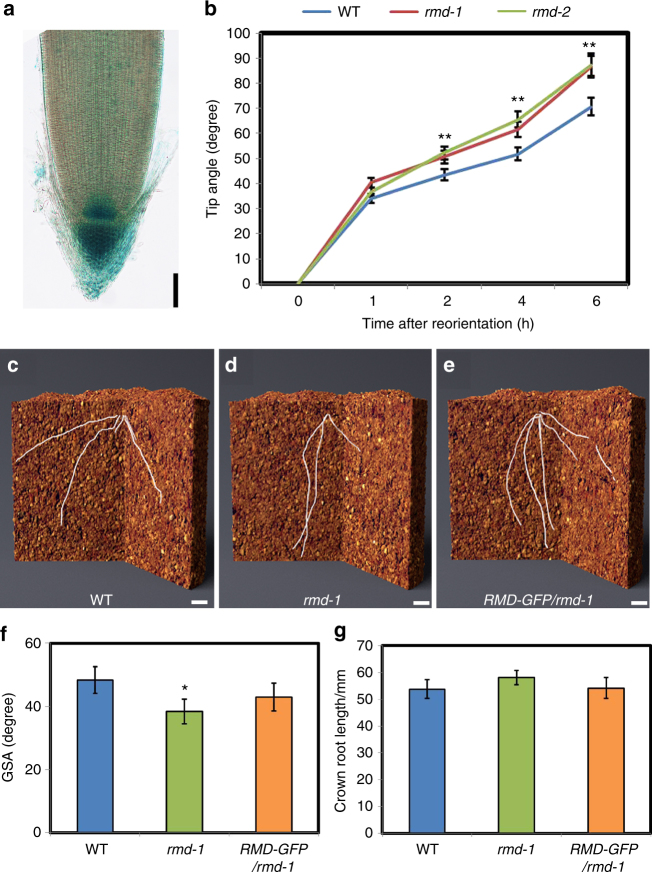
*rmd* exhibits hypersensitive gravitropism and steeper root growth angle. **a** GUS staining of the primary root tips of the seedlings carrying *proRMD::GUS*. Bar, 200 μm. **b** Primary root tip angle over time following a 90° reorientation. Error bars are ±SE, *n* = 3 independent biological replicates from one fully rescued lines with 12 roots analyzed in each assay. Two asterisks show significant difference (*P* *<* 0.01 from Student’s *t*-test). **c** The image of root system architecture (RSA) of WT showing the angle representing the mean angle of the treated sample in soil. Plants were first germinated in water and dark for 3 days, then grown in soil for 7 days. Bar, 10 mm. **d** The image of RSA of *rmd-1* showing the angle representing the mean angle of the treated sample in soil. Bar, 10 mm. **e** The image of RSA of *RMD-GFP*/*rmd-1* showing the angle representing the mean angle of the treated sample in soil. Bar, 10 mm. **f** The GSA analysis of crown roots in WT, *rmd-1*, and rescued line. Error bars are ±SE, *n* = 3 independent replicates with 10 crown roots analyzed in each assay (*P* < 0.05 from Student’s *t*-test). **g** Crown roots length in WT, *rmd-1*, and rescued line. Error bars are ±SE, *n* = 3 independent replicates with 10 crown roots analyzed in each assay

Columella cells at the root tip function as specialized gravity-sensing cells^18,19^. The *RMD* gene is expressed in rice root columella cells (Fig. 1a) and *rmd* mutants exhibit altered shoot and wavy root growth behaviours^20^, prompting us to test whether this gene regulates root gravitropism. Wild-type (WT), *rmd-1*, and *rmd-2* seedling roots were placed horizontally and their rates of bending determined. Intriguingly, *rmd-1* and *rmd-2* mutant roots exhibited faster rates of curvature after the 90° reorientation compared with WT (Fig. 1b and Supplementary Fig. 1a, b and Supplementary Movie 1), indicating a stronger gravitropic response. Similarly, crown roots of 10-day-old *rmd-1* seedlings re-oriented 90° also showed enhanced gravitropism (Supplementary Fig. 1c–g and Supplementary Movie 2). In contrast, lateral roots did not alter their angle following reorientation. Hence, RMD appears to function to dampen primary and crown root gravitropic responses.

To test the effects of *rmd-1* on root growth angle, we re-orientated seedling roots at 30°, 45°, 60°, and 90°, then monitored WT and mutant crown root angle response over 36 h (Supplementary Fig. 1c–g). Our analysis revealed that *rmd-1* crown roots exhibited an enhanced rate of reorientation at every angle tested compared to WT, consistent with the mutant’s faster gravitropic response. Importantly, both WT and *rmd-1* crown roots returned to a vertical direction of growth with respect to gravity, with *rmd*-*1* roots simply reaching a plateau of no further change in tip angle much earlier than WT (Supplementary Fig. 1c–g). These data suggest that rice crown roots are being actively maintained at inherent GSAs and further, that *rmd-1* mutants retain the capacity to robustly maintain crown root GSA.

To investigate whether RMD also affects root angle in soil grown rice plants, we used X-ray microcomputed tomography (μCT) to non-destructively image the root system of WT and *rmd-1* in three dimensions. CT imaging revealed that WT crown roots exhibited smaller root angles near the soil surface (Fig. 1c, f), while *rmd-1* had a steeper root system with larger root angles (Fig. 1d, f). In contrast, crown root length is normal in *rmd-1* (Fig. 1g). The *rmd-1* root angle defect could be rescued by expressing a functional RMD-GFP transgene (Fig. 1e, f). Hence, *RMD* negatively regulates rice crown root angle.

### RMD expression in gravity-sensing cells modulates root angle

We next investigated how RMD may control rice crown root angle. Given that *rmd* disrupts both actin filaments (AFs) and microtubules^20^, we initially attempted to mimic its enhanced gravitropism phenotype by treating WT roots with either the actin polymerization inhibitor LatB or the microtubule depolymerization chemical oryzalin. After 6-h gravitropism treatment, only WT roots treated with LatB showed enhanced gravitropism (Supplementary Fig. 2a, b), consistent with previous results^21–24^. Furthermore, we found that actin depolymerization inhibitor Jasplakinolide (Jasp)-treated roots exhibited the opposite phenotype after 6-h gravitropism treatment and the combined LatB and Jasp-treated roots displayed similar phenotype to WT (Supplementary Fig. 2c–g). These results suggest that RMD affects root gravitropism via an actin-related regulatory function.

**Fig. 2:**
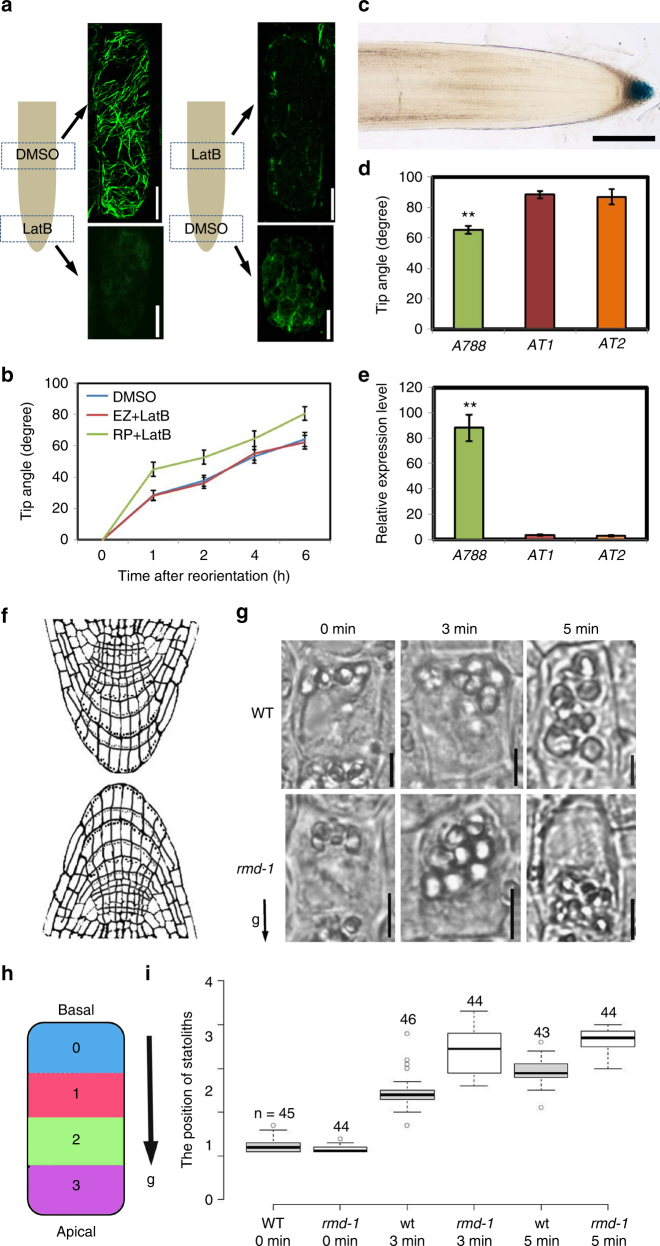
Enhanced gravity-sensing in *rmd* causes hypersensitive root gravitropism. **a** Specific disruption AFs in primary root tip or elongation zone by 1 µM LatB treatment. Bars, 10 μm. **b** Change in root tip angle of the LatB-treated primary roots at denoted times after gravitropism. Error bars are mean ± SE, *n* = 3 three independent biological replicates with at least 18 roots analyzed for each assay. **c** Gus signal was specially observed in columella cells in enhancer trap line *A788*. Bar, 200 μm. **d** Change in root tip angle of *A788*, *RMD-AT1*, and *RMD-AT2* after 6-h gravistimulation. Error bars mean ± SE, *n* = 3 three independent biological replicates with at least 20 roots analyzed for each assays. **e** Transcription analysis of *RMD* expression level in the primary root tips of *A788*, *RMD-AT1,* and *RMD-AT2*. Error bars mean ± SE, *n* = 3 three independent biological replicates from one representative transgenic line. **f** The schematic illustration of the experiment. **g** The representative images of primary roots columella cells after gravistimulation in WT and *rmd-1*. Bars, 10 μm. **h**, **i** Box plots of the comparison of the statoliths along the direction of gravity in columella cells between WT and *rmd-1*. Every dot represents the average position for one columella from different roots

Root tip and elongation zones are known to be responsible for gravity-sensing and gravitropic bending responses, respectively^25^. To reveal whether the *rmd* defect arose from defects in gravity-sensing and/or gravitropic response, we selectively disrupted AFs either within the root tip or elongation zones by locally applying LatB (Fig. 2a). Following local LatB treatments, roots only showed an enhanced gravitropic response after altering AFs in the root tip (Fig. 2b). Hence, RMD affects root gravitropism via AFs within root tip cells.

To directly demonstrate that RMD controls root angle through its expression domain in columella cells at the root tip, we employed the primary root columella specific rice GAL4 enhancer trap line *A788* (Fig. 2c) to create a tissue-specific knock-down line^26,27^. To specifically reduce *RMD* mRNA levels in root columella cells, we adopted an antisense RNA strategy via inserting two independent partial and reverse *RMD* cDNA after the upstream activation sequence that is bound by the columella expressed GAL4 transcription factor in rice line *A788*. Transgenic *A788* *>* *>* *asRMD* lines with reduced *RMD* root tip expression exhibited an enhanced root gravitropic response (Fig. 2c–e). Collectively, our inhibitor and transgenic studies demonstrate that RMD modulates rice root angle via its expression domain in gravity-sensing columella cells.

### RMD functions to link statoliths and AFs

Columella cells sense the direction of gravity through sedimentation of specialized starch-filled plastids termed statoliths^18^. Given that size and number of statoliths are two factors determining the rate of sedimentation, we quantified these properties in both primary and crown roots of WT and *rmd-1*, but no difference was detected (Supplementary Fig. 2h–k). Nevertheless, statolith sedimentation occurred much faster in *rmd-1* than WT following a gravity stimulus (Fig. 2f–i and Supplementary Fig. 3a, b). LatB-treated roots showed a similar phenotype to *rmd-1*, whereas Jasp had the opposite effect (Supplementary Fig. 2i, m). Our results suggest that RMD functions to buffer statolith movement, resulting in a stronger gravitropic response in *rmd-1*.

**Fig. 3:**
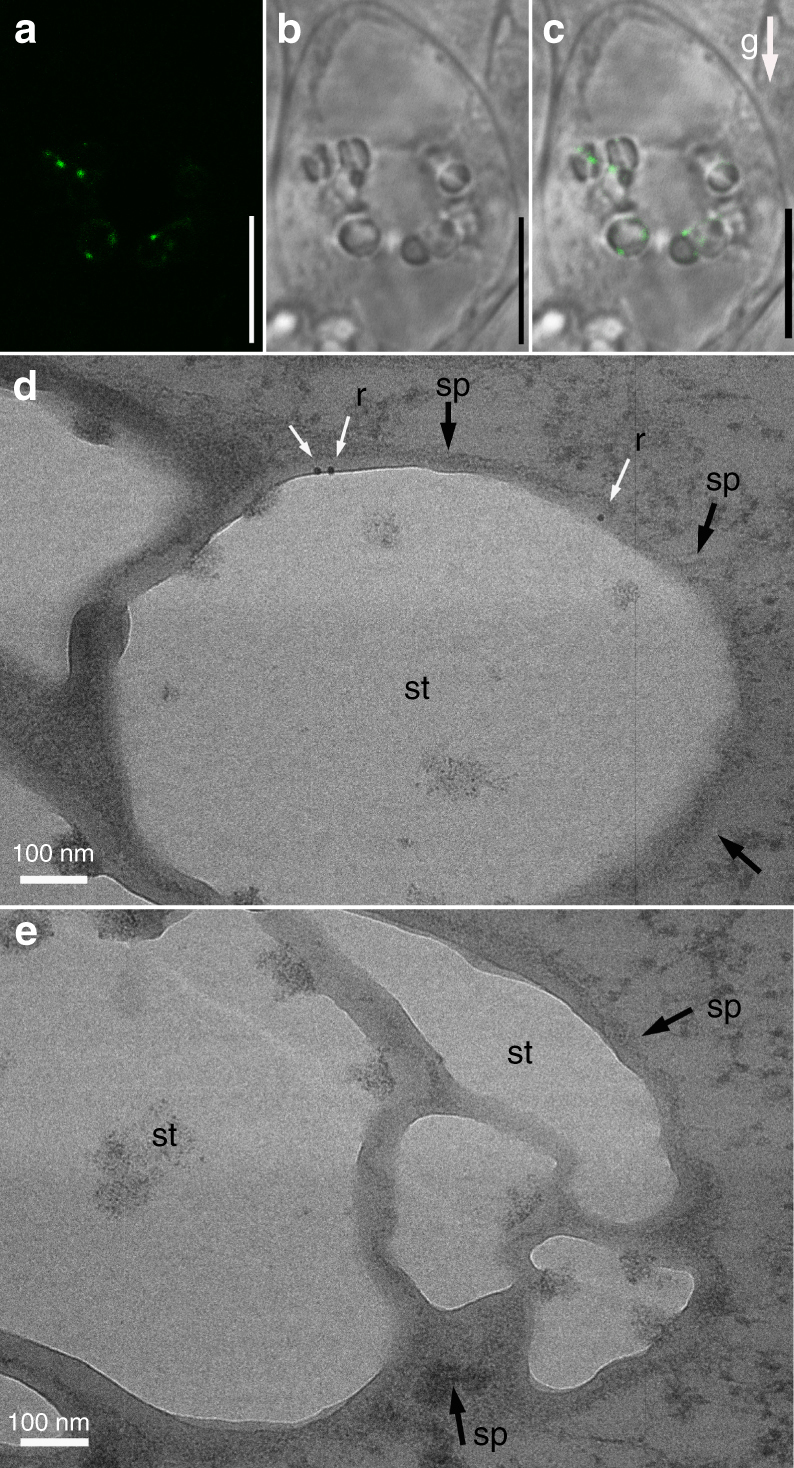
RMD is localized on the surface of statoliths in columella cells. **a**–**c** RMD-GFP co-localizes with statoliths in root columella cells. Bars, 5 μm. **d** Immunogold labeling of rice root columella cells using anti-GFP (10-nm gold-conjugated rabbit secondary antibodies, white arrow; statoliths, black arrow) antibodies in *RMD-GFP/rmd-1* lines. There are 3 ± 0.333 gold particles detected on the surface of each statolith from the TEM section, 3 biological replicates each with 12 slices analyzed. Bar, 100 nm. sp: envelope of statoliths, st: starch grain, r: RMD-GFP. **e** Immunogold labeling of rice root columella cells by using anti-GFP antibody (statoliths, black arrow) in WT. Bar, 100 nm. sp: envelope of statoliths, st: starch grain

Given that RMD functions to regulate AFs dynamics^20,28^, we monitored their behaviour in columella cells. Labelling revealed that AFs form bright ring-like structures around wild-type statoliths, but the AF signal was reduced in *rmd-1* (Supplementary Fig. 3c–e). The remaining AF fluorescence signal surrounding *rmd* statoliths is explained by functional redundancy with other members of the formin family as we observed 9 of the 16 formin genes are expressed in rice root tissues, overlapping with RMD^28^. Confocal-based imaging of transgenic plants expressing *ProRMD*-*RMD*_*CDS*_*-GFP* in *rmd-1* revealed that RMD-GFP was localized on the surface of statoliths in columella cells (Fig. 3a), which was confirmed by high-resolution immunogold-TEM using anti-GFP antibody (Fig. 3b, c). Given that statoliths and chloroplasts are the derivatives of plastids, the observation of RMD localization on the surface of statoliths is consistent with our previous report that RMD is localized on the outer envelope membrane of chloroplasts via its PTEN domain in leaves^20^. Our results suggest that RMD functions to link statoliths and AFs.

### Lateral auxin gradient formation is faster in *rmd* roots

Statolith sedimentation has recently been demonstrated to induce a lateral auxin gradient that is required to trigger a root bending response^29^. To observe whether faster statolith sedimentation in *rmd* alters the mutant’s auxin response dynamics, the auxin response reporter *DR5::3XVENUS-N7*^30^ was transformed into WT rice and then crossed into the *rmd-2* mutant background. Confocal images revealed that the *DR5::3XVENUS-N7* reporter was detected in vascular, columella, and lateral root-cap cells of both WT and *rmd-2* (Supplementary Fig. 4a). Following a gravity stimulus, the asymmetric distribution of *DR5::3XVENUS-N7* was observed much earlier in primary and crown roots of *rmd-2* compared with the WT (Supplementary Fig. 4a). Quantification of the ratio of the reporter in root cells on the top versus bottom revealed the lateral auxin response gradient forms in *rmd-2* primary and crown roots 2-h after a gravity stimulus, whereas the auxin response gradient in wild type required 4-h treatment (Supplementary Fig. 4b, c).

The more rapid and robust gravity-induced auxin response of *rmd* is likely to be reflected in faster differential cell growth and enhanced root gravitropic curvature. To quantify the effects of auxin on root curvature, we calculated the cell length of upper and lower sides of the root at several time points following a gravity stimulus. While no difference was observed between WT and *rmd-1* before a gravity stimulus (Supplementary Fig. 4d–f), the cell length on the upper side of *rmd-1* roots was longer compared to WT from 30 min until 6 h after a gravity stimulus, while the cell length on the lower side of *rmd-1* roots appeared shorter compared to WT, suggesting that the coordinated activity of upper side and lower side cell elongation leading to enhanced gravitropism of *rmd-1* (Supplementary Fig. 4d–f). Hence, *rmd* roots exhibit a more rapid gravity-induced auxin response than WT, triggering faster differential cell growth and a higher rate of gravitropic curvature.

### RMD is required for phosphate-dependent change in root angle

**Fig. 4:**
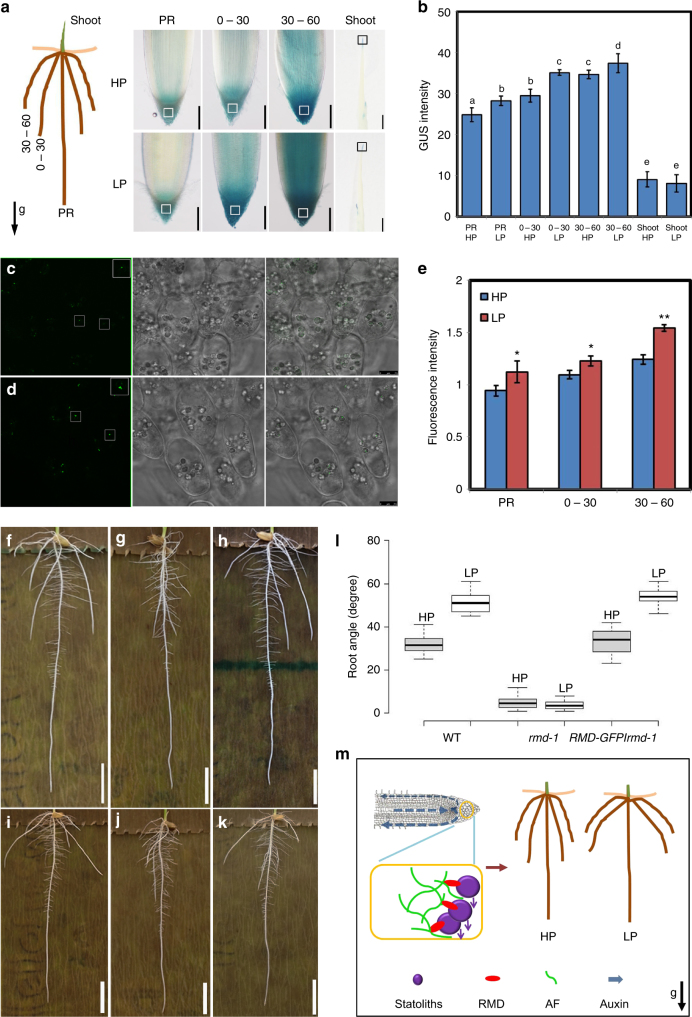
RSA of *rmd-1* showed no response to high or low P conditions. **a** Representative sections of GUS staining of primary root (PR), 0–30° crown root (0–30), 30–60° crown roots (30–60) and shoots in HP and LP conditions. Bar, 100 μm and 1 mm in roots and shoots. **b** GUS staining analysis of *proRMD::GUS* in the boxed region under HP and LP conditions. Error bars mean ± SD, *n* = 15. The letters in (**b**) were used to indicate the difference between each other *P* < 0.05 in (a, b) and (c, d) from Student’s *t*-test; *P* < 0.01 in (a, c), (b, c), (b, d) and (e, f) from Student’s *t*-test. **c** The representative images of *RMD-GFP/rmd-1* in columella cells of 30°–60° crown roots in HP. Bar, 7.5 μm. **d** The representative images of *RMD-GFP/rmd-1* in columella cells of primary roots, 0–30° crown roots, and 30–60° crown roots in LP. Bar, 7.5 μm. **e** The relative fluorescence intensity of HP and LP in the boxed region in primary roots and crown roots. Error bars means ± SE, *n* = three independent assays with 13 cells from different roots analyzed in each assay. Student’s *t*-test: *P < 0.05; **P < 0.01. **f**–**h** From left to right, representative images of WT, *rmd-1*, and rescued lines in HP. Bars, 1 cm. **i**–**k** From left to right, representative images of WT, *rmd-1*, and rescued lines in LP. Bars, 1 cm. **l** Box plots of the root angle of WT, *rmd-1*, and rescued lines grown in high P (HP) or low P (LP) conditions, *n* = 24. In HP, *P* < 0.01 (WT, *rmd-1*), *P* < 0.01 (*rmd-1*, *RMD-GFP*/*rmd-1*), *P* > 0.05 (WT, *RMD-GFP*/*rmd-1*) form Student’s *t*-test. In LP, *P* < 0.01 (WT, *rmd-1*), *P* < 0.01 (*rmd-1*, *RMD-GFP*/*rmd-1*), *P* > 0.05 (WT, *RMD-GFP*/*rmd-1*) from Student’s *t*-test. **m** A proposed model for RMD dependent regulation of RSA by phosphate. RMD links amyloplasts (statoliths) and AFs in root columella cells which is essential for RSA adaptation to different phosphate conditions through fine-tuning gravitropism

A key question is why does RMD function to buffer the root gravitropic response? Shallow root angles are reported to be a developmental adaptation to low phosphate (LP) availability^31^. To address this point, we grew rice seedlings in different phosphate concentrations and observed that crown root angle in WT was negatively correlated with phosphate availability (Supplementary Fig. 5a–e). To understand whether RMD is required for phosphate-dependent changes in root angle, we investigated the impact of disrupting RMD function under high phosphate (HP) and LP conditions. WT roots fully re-orientated in about 8 h under HP conditions, while it took 10 h under LP conditions (Supplementary Fig. 6a, b). In contrast, *rmd-1* roots showed no obvious difference in response under HP versus LP treatment. Strikingly, primary root angle of columella tissue-specific RMD RNAi lines *RMD-AT1* and *RMD*-*AT2* and plants treated by LatB- and SMIFH2 (Small Molecule Inhibitor of Formin (FH2)-treated samples)^32^ were no longer sensitive to external phosphate availability (Fig. 4 and Supplementary Fig. 5f–i and Supplementary Fig. 6). In contrast, crown roots of WT and *RMD-GFP/rmd-1* rescue lines exhibited shallower angles under LP versus HP conditions (Fig. 4f–k and Supplementary Fig. 5f, g).

Does RMD contribute to phosphate uptake efficiency and shoot biomass? ICP-MS measurements of shoot phosphate revealed reduced accumulation of this nutrient in *rmd-1* when grown in different soil phosphate concentrations compared to WT and *RMD-GFP/rmd-1* lines, particularly under split phosphate conditions that mimic normal soil conditions (i.e. HP upper soil; LP lower soil profile) (Supplementary Fig. 7a–c), similar results were observed with shoot biomass (Supplementary Fig. 7d–f). Hence, RMD-dependent buffering of crown root angle change appears to function to improve rice phosphate uptake efficiency and shoot biomass.

### RMD expression positively correlates with crown root angle

How does RMD modify crown root growth angle in a phosphate-dependent manner? Quantification of transcript and protein levels revealed that RMD was more abundant under LP (versus HP) conditions in the root (Fig. 4a–e). In contrast, RMD transcript levels did not change in response to different temperatures, pH values or drought stress conditions (Supplementary Fig. 8a–c). The *RMD* promoter contains 15 auxin (AuxRE) and 4 phosphate (PHR1-like) response motifs within the 3000-bp region upstream of the start codon. However, we detect no obvious signal difference of the auxin response reporter *DR5::3XVENUS-N7* in WT columella cells under LP versus HP, while phosphate-starvation makers *Phosphate Transporter 2* (*PT2*) and *Phosphate Transporter 6* (*PT6*) were upregulated (Supplementary Fig. 8e–g). We therefore reason that upregulated RMD expression in columella cells under LP results from the response to low phosphate (rather than auxin signaling). Moreover, *proRMD::GUS* and *RMD-GFP/rmd-1* reporters are higher in shallow crown roots versus steeper crown roots and primary roots, especially under LP conditions (Fig. 4a–e). Consistent with RMD up-regulation, the fluorescence intensity of ring-like AFs around columella statoliths is enhanced under LP (Supplementary Fig. 9a–c). In addition, the sedimentation of WT statoliths was slower under LP than HP, whereas no change was observed in *rmd-1* (Supplementary Fig. 9d, e). The dynamics of lateral auxin gradient formation (detected using *DR5::3XVENUS-N7*) being enhanced in primary roots and crown roots under HP (Supplementary Fig. 10a–c) and in an RMD-dependent manner (Supplementary Fig. 10d–f). Our results revealed that RMD expression level is positively correlated with the formation of shallow crown root angles. Interestingly, variation in RMD levels under high and low phosphate does not affect the growth angle of the primary root, indicating that RMD-mediated responses to soil phosphate are restricted to roots with non-vertical GSAs. This interpretation would be consistent with the idea that root branches and primary roots are fundamentally different in terms of GSA control despite the many apparent similarities in the machinery of gravity perception and gravitropic response between these root classes.

## Discussion

We report that the actin-binding protein RMD controls root growth angle in response to external phosphate. We propose a model for RMD action (Fig. 4m), where under LP, higher RMD levels in columella cells promote stronger interactions between AFs and statoliths that delay their sedimentation, resulting in less robust auxin-driven root gravitropic response and shallower crown root angle. In contrast, lower RMD levels ultimately result in a steeper crown root angle under HP. RMD therefore appears to function to buffer gravitropic signalling in response to external phosphate levels. While the current data show that the adaptive root angle response is RMD dependent, further work would be required to determine the extent to which up-regulation of RMD at the transcript level is responsible for this. Our work provides new mechanistic insights into how plants regulate key components of the gravitropic machinery to adapt their root systems architecture in response to soil nutrient availability and provide new molecular targets for plant breeding.

## Methods

### Plant materials and growth conditions

The genetic background of rice cultivars were 9522 (*Oryza sativa* L ssp *Japonica*) and Zhonghua11 (ZH11) (*Oryza sativa* L ssp *Japonica*) which grow well in the filed in Shanghai. The genetic background of *A788* is ZH11 and others are 9522. Two alleles *rmd-1* and *rmd-2* with the null mutations at RMD mutants were generated by ^60^Co-ray treatment from 9522. *rmd-1*has a T-to-C transition and a four-nucleotide (AAGG) deletion in the 11th exon of RMD gene, causing the premature termination of the protein at the 1465th amino acid. *rmd-2* contains a four-nucleotide in the fourth exon of *RMD*, causing the premature termination at the 392nd amino acid^20^. Rice plants were cultured in Shanghai (30_N, 121_E) in the summer and Sanya (18_N, 109_E), China, in the winter seasons. The seedlings were cultured in a light incubator with 18 h light/6 h dark at 28 °C.

### Gravitropism analysis

The rice seeds were germinated under constant dark at 28 °C for 3 days and then transferred on 96-well plates without bottom floating on water with 18-h light and 6-h dark at 28 °C. The 3-day-old seedlings were placed on 0.9% agar in normal condition for 1 h, and then were placed horizontally. Photographs were taken automatically every 5 min by a digital camera (Canon) under the control of ZoomBrower EX software (Canon). The primary root curvature was defined as the angle formed between the growing direction of the apex and the horizontal base line and was measured on the digital images using ImageJ (http://rsb.info.nih.gov/ij/). Root gravitropism movies were performed as described by Wells et al.^33^ using automated image acquisition. GSA of crown roots was measured at the insertion site between crown root and primary root with reference to the gravity vector. For the gravitropism analysis of crown roots at different angles, the seeds were germinated for 3 days and grown in the paper bags for 7 days, the whole root systems were put on the surface of 1% agar plate at different angles and finally the plates were put up vertically. The curvature angle of the crown roots were analyzed at the bending sites with the reference to the horizontal direction via ImageJ.

### RT-PCR and plasmid construction

Total RNA was extracted using TRIzol reagent (Invitrogen) according to the instruction of the manufacturer. One microgram RNA was used to synthesize the first-strand cDNA using the Rever Tra Ace-а-First strand cDNA synthesis kit (TOYOBO). Primers used for RT-qPCR are as follows: ZP190/ZP191 for *RMD*; ZP196/197 and ZP198/199 for *PT2* and *PT6* ^34^; ZP202/ZP203 for *ACTIN*, ZP204/ZP205 for *TUB*. The quantitative RT-PCR (qRT-PCR) analysis was performed as previously described^35^. The samples were picked from the region about from 0 to 3 mm distal from the primary or/and crown root tips of the 4-day-old seedlings. The rice *TUB* and *ACTIN* gene was used as an internal control. The related primers are listed in Supplementary Table 1.

All PCR amplifications were done with KOD DNA polymerases with the recommended annealing temperature and extension time. Sequences were analyzed with Vector NTI11 (Invitrogen). PCR products were recovered with QIAquick^®^ Spin miniprep kit, and DNA midipreps were with the Qiagen TIP-100 kit. The *proRMD::RMD-GFP* reporter construct was constructed via the In-Fusion technology (http://www.clontech.com). For these vectors, more than 15 independent transgenic lines were obtained for each vector. Primers were used as follows: ZP1/ZP2 for *RMD-GFP*. *RMD AT1* and *RMD AT2* vectors were generated from the LIC-based clone technology^36^. Primers were used as follows: ZP44/ZP45 for *RMD AT1* and ZP46/ZP47 for *RMD AT2*.

### Pharmacological treatments

Cytoskeleton disruption drug LatB, Jasp and Oryzalin were purchased from Sigma company (http://www.sigmaaldrich.com/). Four-day-old roots were immersed in 1 µM LatB, 0.5 µM Jasp, and 1 µM oryzalin for 30 min before the following experiments. The treated roots were placed horizontally on the MS medium for 6-h treatment, and then were used for analysis.

For the localized treatment of LatB, the 1 mm filter papers were immersed into the 1 µM LatB for 10 min. After LatB incubation, the filter papers were applied to either the cap or the elongation zone (2.5 mm from the root tip) for 1 h. The treated roots were grown for 30 min before gravitropism treatment and microscope observation.

### F-actin and GUS staining and cell length analysis

The method of F-actin staining for rice root tip was modified from the reported description^37^. The 1.5-cm root tip was incubated in PEM buffer containing 2% (w/v) glycerol (Sigma-Aldrich) and 6.6 µM Alexa Fluor 488-phalloidin/Rhodamine Phalloidin (Invitrogen) staining for 40 min. Stained roots were put on and then were observed via a Laser Scanning Microscope SP8 (Leica).

The plant roots were incubated in 50 mM Na_3_PO_4_ (pH 7.0) that containing 10 mg/mL X-Gluc and 0.02% (v/v) TritonX-100, under dark at 37 °C for 3 h. After staining, the samples were washed with 70% ethanol for 1 h followed by 100% ethanol for 12 h at room temperature. The stained roots were embedded into 5% low melting agar and then sliced into pieces via microtome (Leica VT 1000s). The GUS staining images were taken via a Leica light microscope (M205A) with a CCD. The GUS signal was analyzed via ImageJ (http://rsb.info.nih.gov/ij/).

The 7-day-old seedlings were fixed in 2.5% glutaraldehyde for 20 min under vacuum. The region of cell length measurement was at the site of 2.5 mm from the root tip at 0 h during gravitropism treatment. After the root tip showing gravitropism, the region of cell length measurement was at the bending site. Images were imaged by the light microscope (Nikon) and analysed by the ImageJ.

### Analysis of statoliths in root columella

For the observation of statoliths sedimentation, the method was modified from the previous description^38^. Rice seeds were germinated in water for 3 days consistently under dark. The germinated seeds were transferred on the plates containing 0.6% agar and grow in 18 h light/6 h dark for 4 days. The samples were put upside down for 0, 3, and 5 min before immediately fixed in 10% (v/v) formaldehyde, 5% (v/v) acetic acid, and 50% (v/v) ethanol in 1 mL tubes under vacuum. During the process, the roots that did not grow upright were carefully excluded from the samples. After fixation, the fixed roots were dehydrated via a series of ethanol (50%, 40%, 30%, 20%, and 10%) washed for 10 min and embedded in poly (ethylene glycerol) disterate (Sigma-Aldrich). The sections (7 μm) were dewaxed in ethanol and then observed under a fluorescence confocal microscope (Leica).

### Transmission electron microscopy and Immunogold labelling

Rice seeds were germinated in the water for 3 days under dark and then grown in the paper bag for 7 days. Seven-day-old rice root tips were selected as samples and processed as the modified method from previously described^39^. The samples were fixed in 2.5% glutaraldehyde under medium vacuum at 4 °C for 2 h. The fixed samples were washed by phosphate-buffered saline (PBS) for three times, 15 min for each time. After removing the last wash of PBS, the samples were fixed in 1% (wt/vol) OsO4 at 4 °C for 30 min. The refixed samples were carefully washed by ddH_2_O for three times, each time should last for at least 20 min. After the last wash with ddH_2_O, 10%, 20%, 30%, 50%, 70%, 90% and 100% (vol/vol) ethanol were added into the tube in order; each step was maintained about for 10 min. Infiltrated the samples with 25%, 50%, 75%, and 100% (vol/vol) LR white resin (ProSciTech, cat. no. C023) in ethanol; each step was maintained at least for 8 h at 4 °C with slightly shaking. The fully infiltrated samples were embedded in gelatin capsules (middle size) and cured at 60 °C for 12 h. The prepared ultrathin sections were etched by NaIO_4_ for 10 min with the samples downside, washed by ddH_2_O for 10 min, and repeated for three times. The washed sections were blocked by 1% bovine serum albumin (BSA) for 20 min, incubated in a moist chamber with primary antibody diluted by 1:200 (anti-GFP; Rabbit monoclonal; Abcam ab32146)^40^ for at RT for 3 h. This step was followed by rising and incubated with second antibody conjugated to 10-nm gold particle (labeling RMD-GFP). The second antibody was diluted by 1:100 (Aurion) with 1% BSA and incubated for 2 h. Sections were thoroughly washed with ddH_2_O, stained with filtered uranyl acetate solution for 8 min, and triple lead citrate for 4 min. The stained sections were washed immediately and thoroughly with ddH_2_O. The sections were viewed by 120 kV Biology Transmission Electron Microscope (FEI).

### Soil preparation and ICP-MS analysis

The pots were washed thoroughly with ddH_2_O and dried in the oven. Each pot **(**82 mm diameter × 117 mm height) could contain about 860 g of LP soils (the available phosphate was 8.6 mg/L). For HP condition, 210 mg KH_2_PO_4_ soil was added into 1 kg LP soil and mixed thoroughly. After mixing thoroughly, the soils were dried in the air, mixed thoroughly again (crushed manually to make the soil particle fine), and sieved (<2 mm) for packing in column. For the LP condition, LP soil was mixed with silver sands in 1:3 (1-part LP soil and 3-part silver sand) to make it phosphate-deficient soil. For split P treatment, lower 12.5 cm of column was filled with low P soil and the remaining 4 cm top was filled with high P-containing soil. Before planting, columns were saturated with ddH_2_O water for up to 4 h. To place the seedling’s root inside the soil a small whole was made and the roots were pushed gently inside the soil.

WT, *rmd-1*, *rmd-2*, and *RMD-GFP/rmd-1* seeds were dehusked and ~2/3rd part of the seeds was excised keeping the embryo intact. These cut seeds were sterilized with 50% bleach solution (NaOCl_2_) for 15 min and washed with sterilized water six times and kept for drying in laminar air flow for 20 min. Further, seeds were placed (embryo side up) in 1/2th MS (0.5% phytagel) media for germination at 28 °C in a rice growth chamber (70% humidity and 300 µM/photon/m^2^/s light condition). After, three DAG equally germinated seedlings were planted in LP and HP soils.

After 21 days growth in low, high, and split phosphate soil conditions, shoots of WT, *rmd-1*, *rmd-2*, and *RMD-GFP/rmd-1* lines were harvested and dried in an oven at 45 °C till constant weight (6 days). All dried seedlings were weighed individually on an electronic balance. Four independent replicates comprising each seedling were further used for digestion and subsequent ICP-MS analysis. All samples were acid digested by adding 2 mL conc. HNO_3,_ 1 mL Milli-Q water, and 1 mL H_2_O_2_ and digested for 45 min in an automated multiwave 3000 microwave (Anton Paar GmbH, Graz, Austria) at 2 MPa pressure. To ensure the complete digestion of rice shoots, certified reference material (wheat-flour 1567b) and internal reference control (tomato leaf powder) were also digested along with the samples and blanks. After complete digestion (transparent solution) samples were diluted by adding 6 ml Milli-Q water in the fume cupboard and transferred to a 25 mL universal tube for storage at room temperature. Further, for ICP-MS analysis samples were finally diluted (1:5) with Milli-Q water (2 mL sample:8 mL Milli-Q water) into labelled ICP tubes. Concentration of 28 elements was analyzed by using ICP-MS (ICP-MS; Thermo Fisher Scientific iCAPQ; Thermo Fisher Scientific, Bremen, Germany).

### Abiotic stress treatments

The seeds were immersed in the water for 3 days in the dark conditions. The germinated seeds were transferred into paper bags wrapped with the aluminum foil, containing 15 mL HP, LP, or HP/LP with 0.4 μM LatB/10 μM SMIFH2 and cultured in the light incubator for 7 days before analysis. The nutrient solution for high- and low-phosphate treatment is described in Table S2. For the LatB- and SMIFH2-treated roots, the germinated seeds were grown in LP containing 0.4 μM LatB or 10 μM SMIFH2. The detailed information for the HP and LP is listed in Supplementary Table 2.

For low- and high-temperature treatment, the seeds were germinated in the water for 3 days under dark and the germinated seeds were grown into the paper bags under 28° for 7 days before treatment. The seedlings were transferred to 22°, 28°, and 37° for 6 h before harvest. For low- and high-pH treatment, the germinated seeds were grown in the papers containing the solution with pH 4.9, pH 5.9, and pH 6.9 for 7 days before harvest. For drought stress treatment, the germinated seeds were grown in the paper bags with 15 mL solution for 7 days, and then transferred to new paper bags with 15 mL, 10 mL, and 5 mL solution for 9 h before harvest.

### Root imaging in soil using µCT

The rice seeds were germinated for 3 days in dark conditions and then grown for 7 days in the soil before used for µCT scans. All X-ray µCT scans of rice roots grown in soil were carried out at The Hounsfield Facility, University of Nottingham. PVC columns (7.2 cm Ø × 15 cm length) were filled with a low available P clay loam sub-soil (air dried and sieved to <2 mm) and saturated with dH_2_O overnight. The low P soil was collected from The University of Nottingham farm field site at Bunny, Nottinghamshire, UK (52.86°N, −1.127°W). Soil was sampled by removing the top 40 cm of soil and then collecting the soil from a depth of 40–60 cm. Available soil nutrient levels were 6.2 mg/L P, 97 mg/L K, and 306 mg/L Mg with a soil pH of 7.3. A high P soil was prepared by saturating the soil with Yoshida nutrient media containing 320 µM KH_2_PO_4_ and then puddled to create a ‘paddy’ condition soil maintained at saturation using high P Yoshida solution.

Two pre-germinated seeds were planted to a depth of 5 mm per column and incubated in an environmentally controlled growth room with 18 h-lightness/6h-darkness cycle at a temperature of 28 °C for 8 days. Soil columns were maintained at saturation for the duration of growth period (‘paddy conditions Yoshida nutrient media’). Soil columns were scanned using a GE v|tomex|m 240 X-ray μCT scanner (GE Measurement and Control Systems, http://www.phoenix-xray.com/). Scans were performed in ‘Fast mode’ where single radiograph images are collected as the sample rotates continuously through 360°. A total of 2160 projection images were acquired at 180 kV X-ray energy and 180 μA current, with a detector exposure time of 200 ms. The distance from the X-ray focal spot to the sample (FOD) and the detector (FDD) were 2044.674 and 818.698 mm, respectively, resulting in a volume with a spatial resolution of 50 μm isotropic voxel size. The total scan time for each sample was 7.2 min.

Rice roots were manually identified in the reconstructed µCT data using the polyline tool in VGStudioMAX v2.2 (Volume Graphics, GmbH, Germany). Starting from the seed, roots were individually traced until they reach the column wall. The polyline was extracted and the *xyz* co-ordinates of the points used to fit a spline for a continuous representation. The roots were then sampled at an equi-distance of 5 mm, and the sampled points used to calculate the tangential angles, averaged along the root. Angles were measured between the vertical axis, such that a low angle represents a steep root while a large angle represents a shallow root.

### Data availability

The authors declare that all data supporting the findings of this study are available within the manuscript and its supplementary files are available from the corresponding author upon request.

## Electronic supplementary material

Supplementary Information

Description of Additional Supplementary Files

Supplementary Movie 1

Supplementary Movie 2
