# Supplementary material for: Rice actin binding protein RMD controls crown root angle in response to external phosphate
Source: Nat Commun. 2018 Jun 11;9:2346. doi: 10.1038/s41467-018-04710-x (PMC5995806; doi:10.1038/s41467-018-04710-x)
Supplement: Supplementary file 1 — Supplementary Information [file 41467_2018_4710_MOESM1_ESM.pdf]

Supplementary Information

Rice actin binding protein RMD controls crown root angle in response to external phosphate

Huang et al

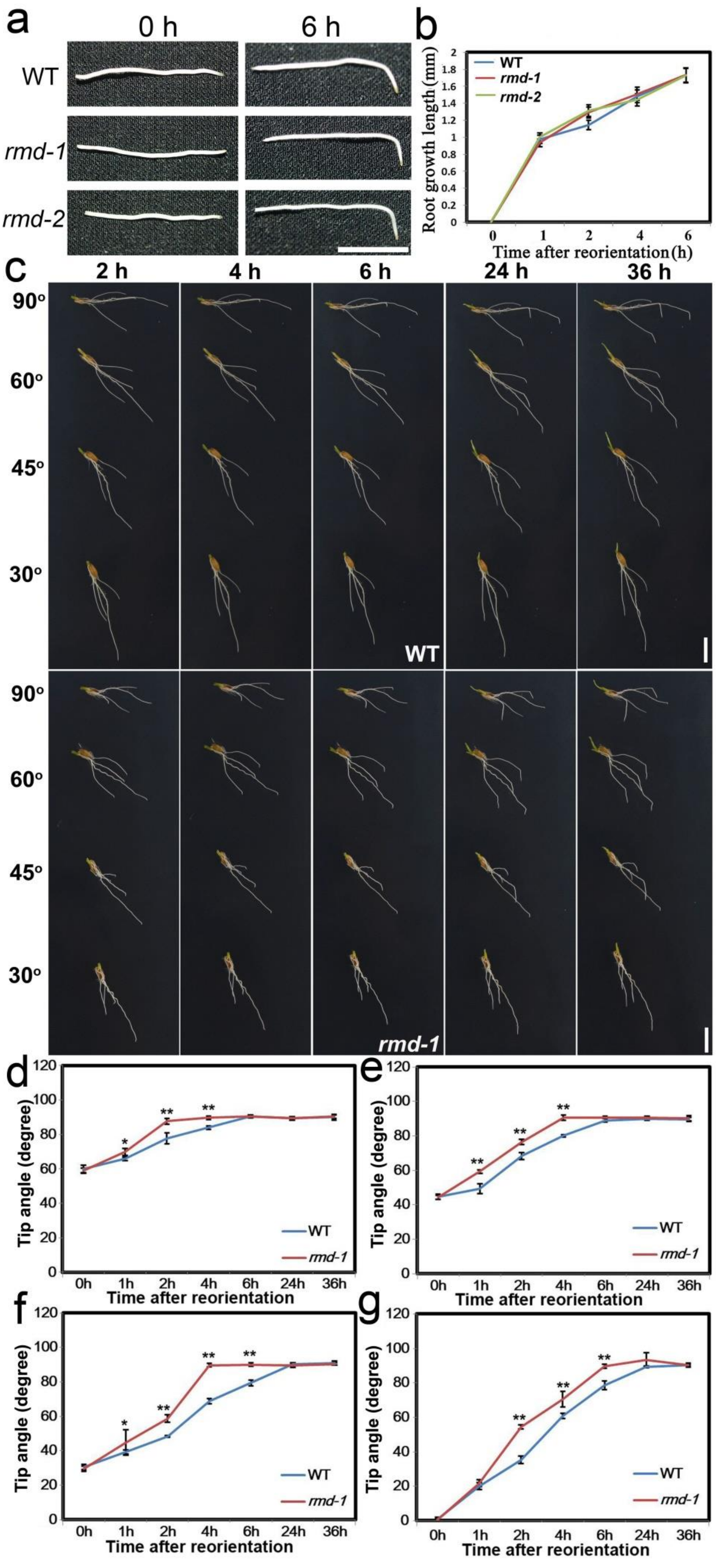
a
0 h
6 h
WT
rmd-1
rmd-2
b
Root growth length (mm)
Time after reorientation (h)
WT
rmd-1
rmd-2
c
2 h
4 h
6 h
24 h
36 h
90°
60°
45°
30°
WT
rmd-1
d
e
f
g
Tip angle (degree)
Time after reorientation
0h
1h
2h
4h
6h
24h
36h

Supplementary Figure 1. Enhanced gravitropism was observed in *rmd* and LatB treated roots. (**a**) The representative primary root images of WT, *rmd-1* and *rmd-2* after a 90° gravistimulation. Bar, 5 mm. (**b**) Root growth length of the primary roots after a 90° gravistimulation. Error bars are ± SE, n = 3 independent experiments with 12 roots analyzed for each assay. (**c)** The representative images of root systems growth after a gravistimulus using different angle in WT and *rmd-1* at 2 h, 4 h, 6 h, 24 h and 36 h, respectively. Bars, 1 cm. (**d-g)** Tip angle degree of crown roots during gravitropism after reoriented 30, 45, 60 and 90 degree. Error bars are ± SE, n = 3 independent biological replicates and each with 12 root system was calculated, one and two asterisks mean $P < 0.05$ and $P < 0.01$ from Student's *t*-test.

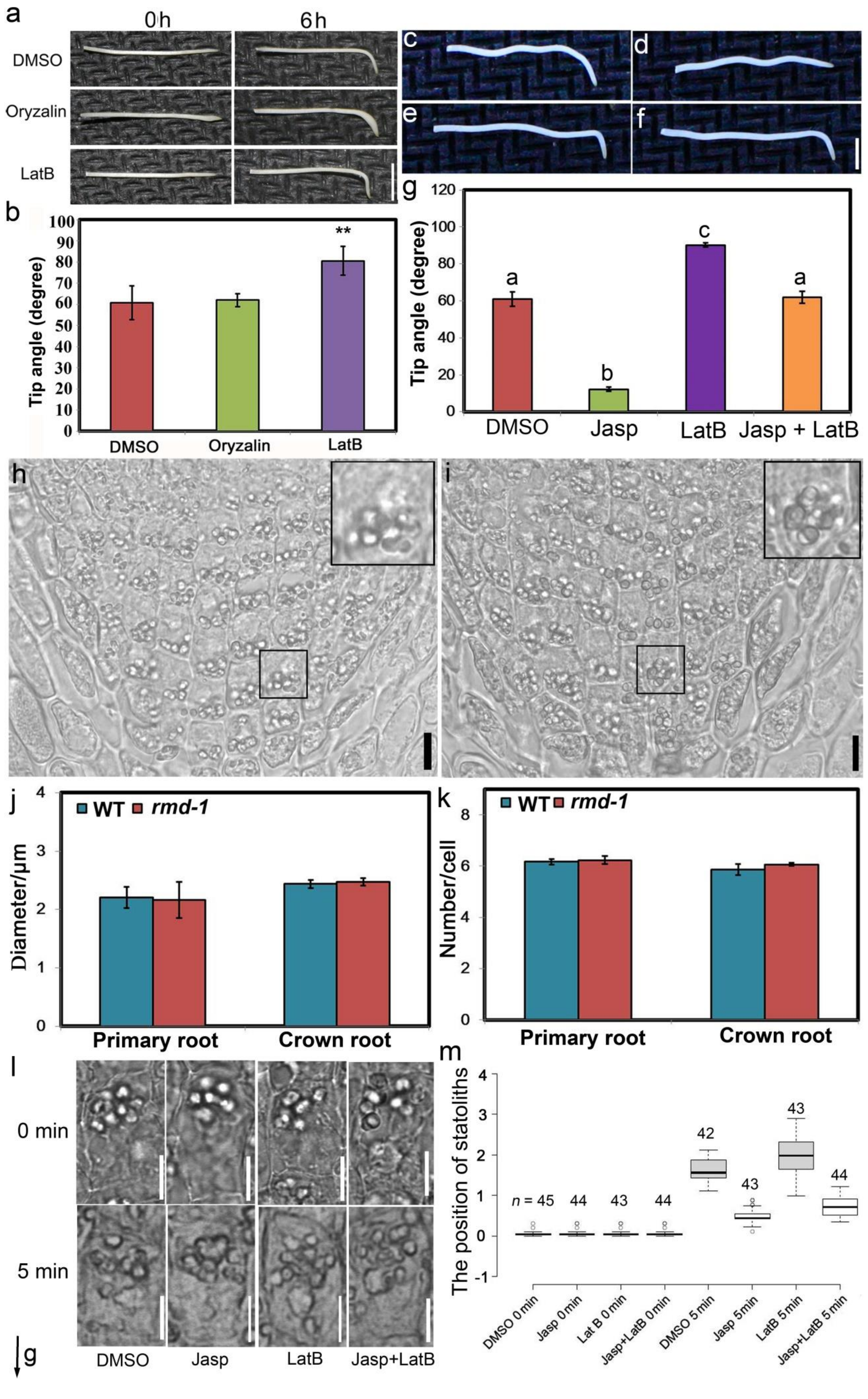
a
0 h
6 h
DMSO
Oryzalin
LatB
c
d
e
f
b
Tip angle (degree)
**
DMSO
Oryzalin
LatB
g
Tip angle (degree)
a
b
c
a
DMSO
Jasp
LatB
Jasp + LatB
h
i
j
WT
rmd-1
Diameter/μm
Primary root
Crown root
k
WT
rmd-1
Number/cell
Primary root
Crown root
l
0 min
5 min
DMSO
Jasp
LatB
Jasp+LatB
g
m
The position of statoliths
n = 45
44
43
44
42
43
43
44
DMSO 0 min
Jasp 0 min
Lat B 0 min
Jasp+LatB 0 min
DMSO 5 min
Jasp 5min
LatB 5 min
Jasp+LatB 5 min

Supplementary Figure 2. LatB and Jasp play antagonistic roles in gravitropism and the sedimentation of statoliths. (a) The representative images of DMSO, Oryzalin and LatB treated primary roots before and after gravistimulation. Bar, 5 mm. (**b**) LatB treated primary roots showed hypersensitive gravitropism. Error bar means ± SE, n = 3 independent experiments with 15 roots analyzed for each assay, two asterisks indicate significant difference ($P < 0.01$ from Student's *t*-test). (**c-f)** The representative images of DMSO, Jasp, LatB and both Jasp and LatB treated primary roots after gravitropism. Bar = 5 mm. **(g)** Effects of LatB and Jasp on primary roots tip angle in gravitropism. Error bar means ± SE, n = 3 independent experiments with 12 roots analyzed for each assay, different characters mean significant difference ($P < 0.01$ from Student's *t*-test). (**h)** The representative image of primary root tip of WT. Bar, 10 μm. (**i)** The representative image of primary root tip of *rmd-1*. Bar, 10 μm. (**j)** The average diameter of statoliths in WT and *rmd-1*. Error bars mean ± SE, n = three independent assays with 12 cells from different roots analyzed in each assay. (**k)** The number of statoliths in each cell in WT and *rmd-1*. Error bars means ± SE, n = three independent assays with 17 cells from different roots analyzed in each assay. (**l)** Representative images of the statoliths in primary roots columella cells during gravitropism. Bars, 1 μm. (**m)** Kinetics comparison of the statoliths along the direction of gravity in columella cells between WT and *rmd-1*. Error bars mean ± SE, n = 3 independent biological replicates with 22 cells from different roots were analyzed for each assays.

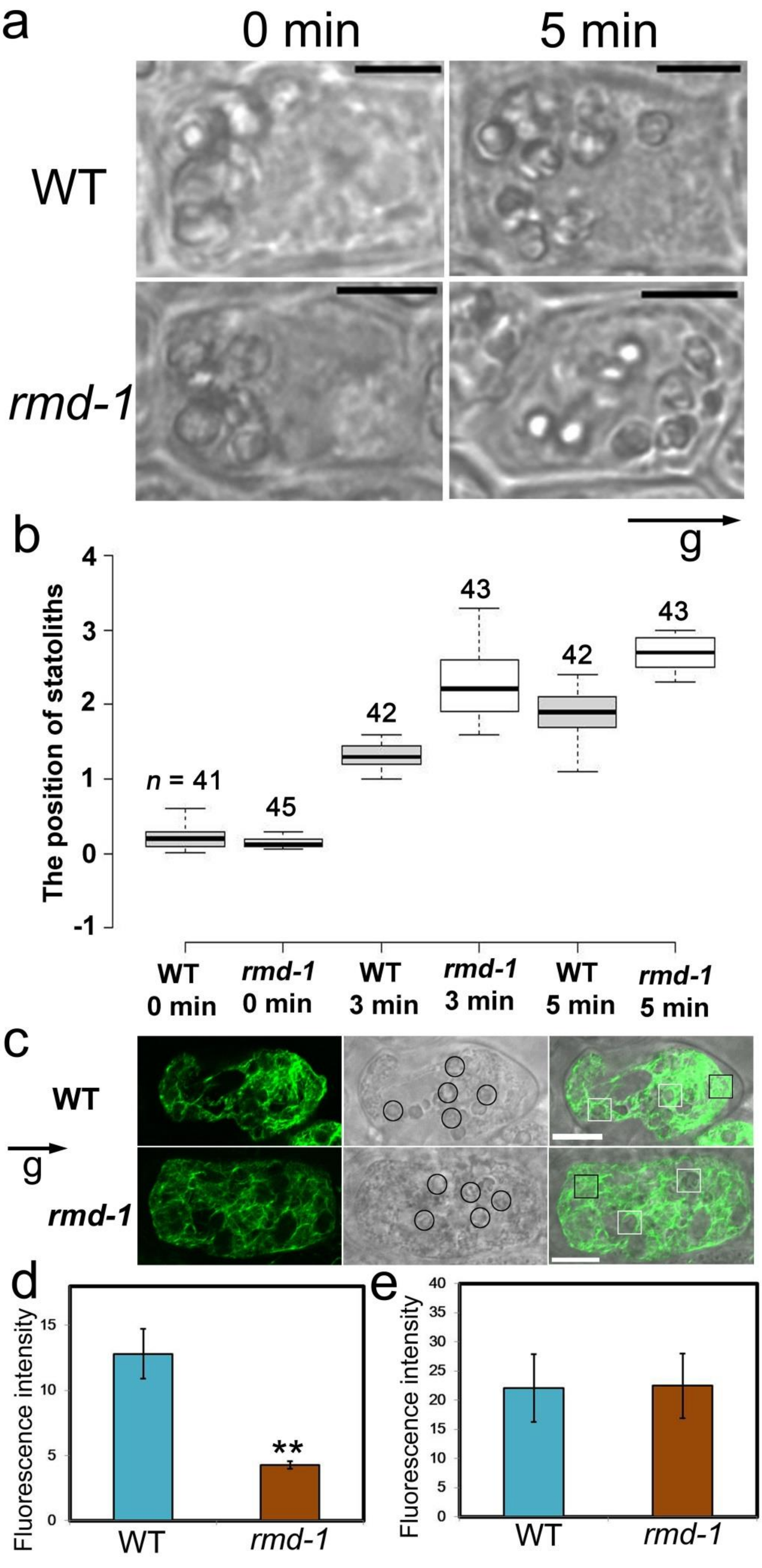
a
0 min
5 min
WT
rmd-1
g
b
The position of statoliths
4
3
2
1
0
-1
n = 41
45
42
43
42
43
WT 0 min
rmd-1 0 min
WT 3 min
rmd-1 3 min
WT 5 min
rmd-1 5 min
c
WT
g
rmd-1
d
Fluorescence intensity
15
10
5
0
**
WT
rmd-1
e
Fluorescence intensity
40
35
30
25
20
15
10
5
0
WT
rmd-1

Supplementary Figure 3. The sedimentation rate of statoliths was faster in crown roots of *rmd-1*. (a) The representative crown roots columella cells in WT and *rmd-1* during gravitropism. Bars, 5 μm. (**b)** The positions of statoliths in WT and *rmd-1*. Error bars mean ± SE, n = 3 three independent biological replicates with 26 cells from different roots analyzed for each assays. (**c)** the representative crown roots images of AFs staining results in columella cells of WT and *rmd-1*. Bars, 5 μm. (**d)** Quantification of AFs fluorescence intensity around statoliths boxed by white region in WT and *rmd-1*. Error bars mean ± SE, n = 3 three independent biological replicates with 12 cells from different roots analyzed for each assays, two asterisks indicate significant difference ($P < 0.01$ from Student's *t*-test). (**e)** Quantification of AFs fluorescence intensity of cytoplasm boxed by black region in WT and *rmd-1*. Error bars mean ± SE, n = 3 three independent biological replicates with 12 cells from different roots analyzed for each assays.

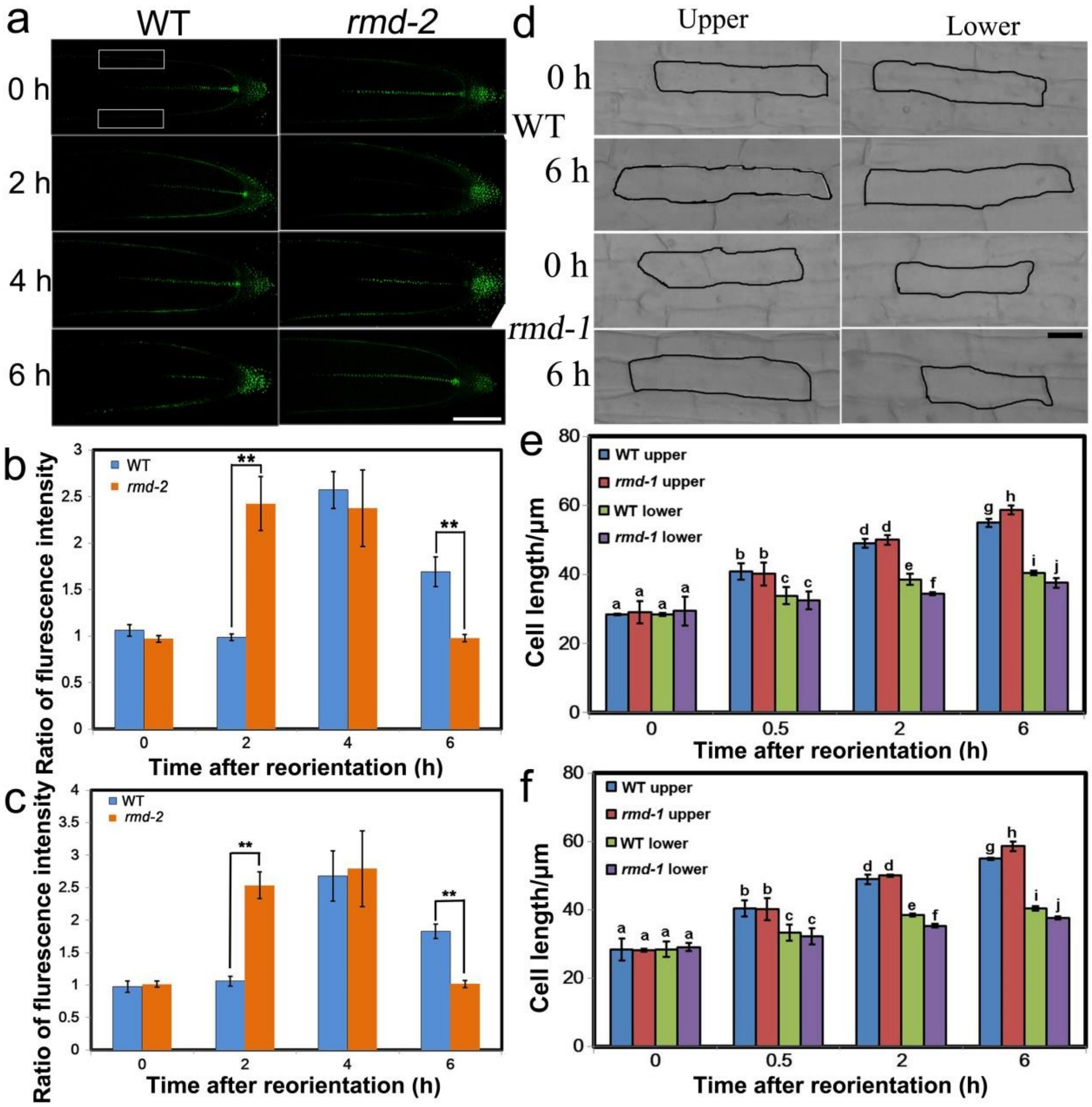

Supplementary Figure 4. The dynamic induction of auxin responsive reporter *DR5:3XVENUS-N7* is faster during root gravitropism in primary and crown roots of *rmd-2* versus WT. (**a**) Fluorescence distribution of *DR5::3XVENUS* after gravistimulation in primary roots of WT and *rmd-2*. The boxed regions were selected as the target for the following statistics. Bar, 200 μm. (**b**) Kinetics of the fluorescence intensity ratio of lower side to upper side after gravitropism in primary roots of WT and *rmd-2*. Error bars are ± SD (n = 15), two asterisks are significant difference ($P <$ 0.01 from Student's *t*-test). In WT, $P >$ 0.05 (0h, 2h), $P <$ 0.01 (2h, 4h), $P <$ 0.01 (4h,

6h), *P* < 0.01, (0h, 6h) from Student's *t*-test. In *rmd-2*, *P* < 0.01 (0h, 2h), *P* > 0.05 (2h, 4h), *P* < 0.01 (4h, 6h), *P* > 0.05 (0h, 6h) from Student's *t*-test. (**c**) Fluorescence distribution of *DR5::3XVENUS-N7* during gravistimulation in crown roots of WT and *rmd-2*. Error bars means ± SE, n = three independent assays conducted with 10 roots analyzed in each assay, two asterisks indicate significant difference (*P* < 0.01 from Student's *t*-test). In WT, *P* > 0.05 (0h, 2h), *P* < 0.01 (2h, 4h), *P* < 0.01 (4h, 6h), *P* < 0.01, (0h, 6h) from Student's *t*-test. In *rmd-2*, *P* < 0.01 (0h, 2h), *P* > 0.05 (2h, 4h), *P* < 0.01 (4h, 6h), *P* > 0.05 (0h, 6h) from Student's *t*-test. (**d**) The representative images of primary roots epidermal cells in WT and *rmd-1* during gravity response. Bar, 10 μm. (**e**) Cell length analysis of primary roots elongation zone during gravitropism. Error bars mean ± SE, n = 3 independent biological replicates with 15 cells from different individual roots in each assay, *P* < 0.01 (a, b), P < 0.01 (b, c), P < 0.01 (e, f), P < 0.01 (g, h), P < 0.01 (i, j), P < 0.05 (c, f), P > 0.05 (e, i) and P > 0.05 (f, j) from Student's *t*-test. (**f**) Cell length analysis of crown roots elongation zone during gravitropism. Error bars mean ± SE, n = 3 independent biological replicates with 15 cells from different roots in each assay, *P* < 0.01 (a, b), P < 0.01 (b, c), P < 0.01 (e, f), P < 0.01 (g, h), P < 0.01 (i, j), P < 0.05 (c, f), P > 0.05 (e, i) and P > 0.05 (f, j) from Student's *t*-test.

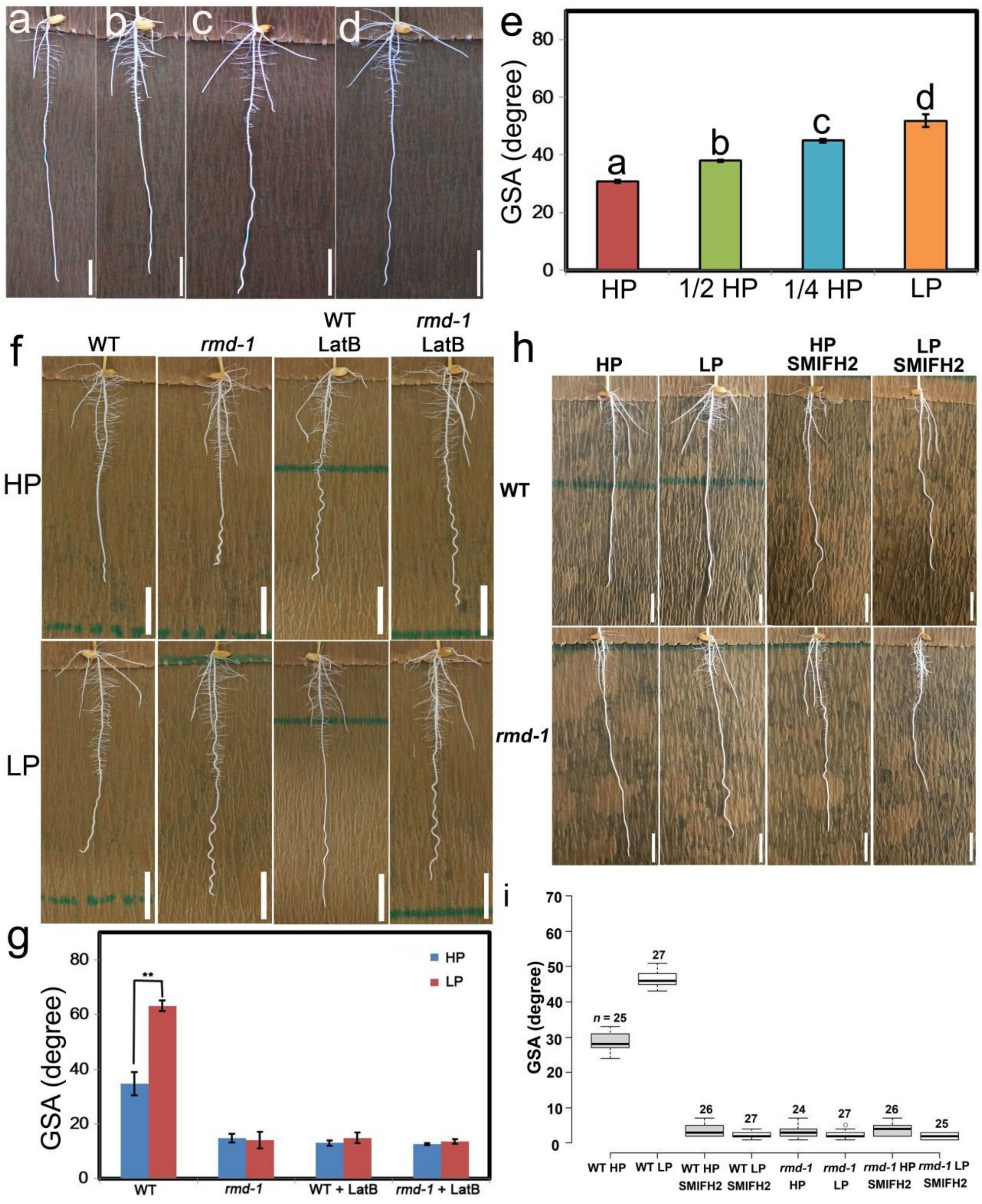

Supplementary Figure 5. LatB- and SMIFH2- treated roots phenocopy phosphate insensitive GSA phenotype of *rmd-1*. (a-d) The representative images of wild-type rice root system after 7-day growth in HP, 1/2 HP, 1/4 HP and LP medium. Bars, 1 cm. **(e)** GSA of crown roots in HP, 1/2 HP, 1/4 HP and LP. Error bars mean ± SE, three independent biological replicates with 14 roots analyzed in each assay, The letters a/b/c/d in (e) are used to indicate the differences between each other, different

characters mean significant difference (*P* < 0.01 from Student's *t*-test). **(f)**, The representative images of WT, *rmd-1* and 0.4 μM LatB-treated root system after 7-day growth in HP and LP. Bars, 1 cm. **(g)** GSA of WT, *rmd-1* and 0.4 μM LatB-treated root system in HP and LP conditions. Error bars mean ± SE, n = three independent biological replicates with 13 root systems were analyzed in each assay, two asterisks mean significant difference (*P* < 0.01 from Student's *t*-test). In HP, *P* < 0.01 (WT, *rmd-1*), *P* < 0.01 (WT, LatB), *P* > 0.05 (*rmd-1*, LatB) form Student's *t*-test. In HP, *P* < 0.01 (WT, *rmd-1*), *P* < 0.01 (WT, LatB), *P* > 0.05 (*rmd-1*, LatB) form Student's *t*-test. **(h)** The representative images of WT, *rmd-1* and 10 μM SMIFH2-treated root system after 7-day growth in HP and LP. Bars, 1 cm. **(i)** GSA of WT, *rmd-1* and 10 μM SMIFH2-treated roots in HP and LP. In WT, *P* < 0.01 (HP, LP), *P* < 0.01 (HP, HP + SMIFH2), *P* > 0.05 (HP + SMIFH2, LP + SMIFH2) from Student's *t*-test; In *rmd-1*, *P* > 0.05 (HP, LP), *P* > 0.05 (HP, HP + SMIFH2), *P* > 0.05 (HP + SMIFH2, LP + SMIFH2) from Student's *t*-test

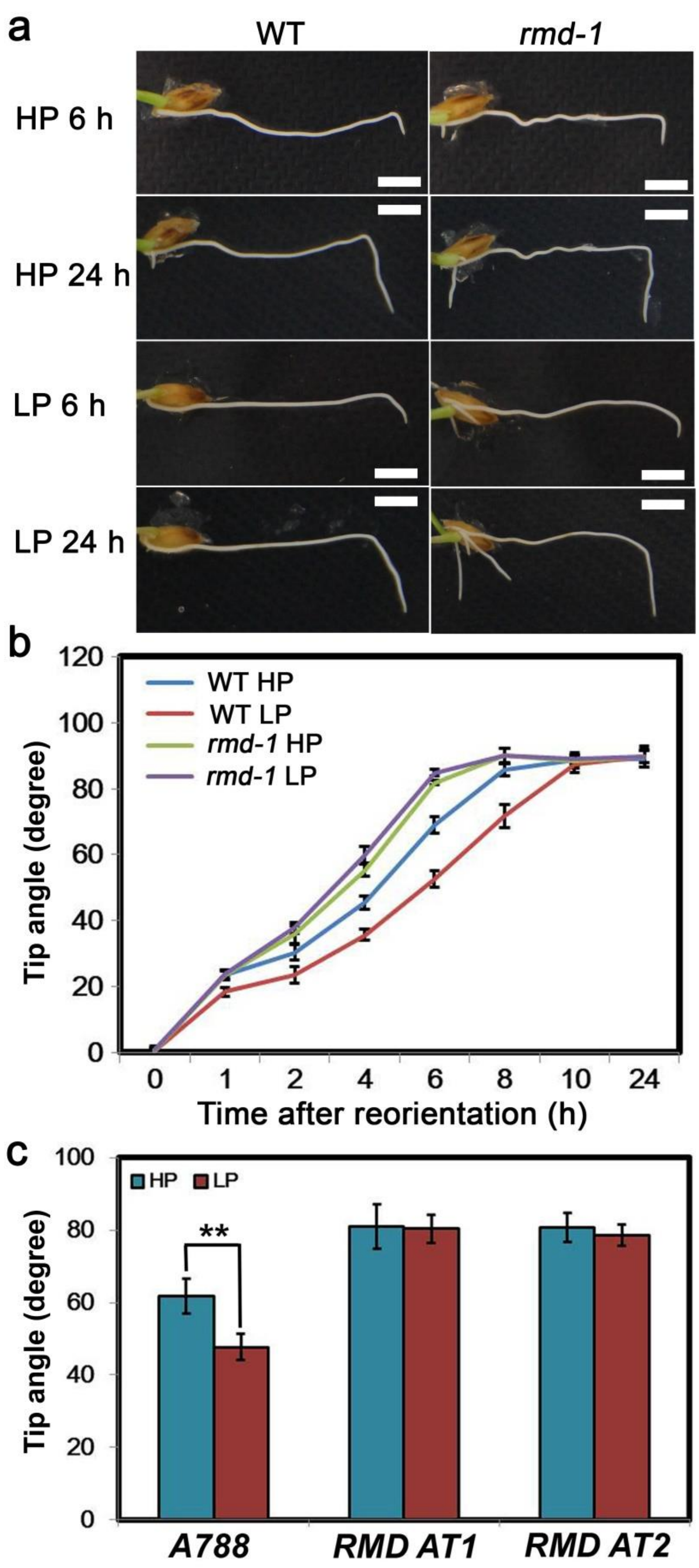

Supplementary Figure 6. Knockdown of *RMD* expression level in columella cells showed insensitive gravitropic response to phosphate availability. (a) The representative images of WT and *rmd-1* after 24-h gravitropic response in HP and LP.

Bars, 5 mm. (**b)** Tip angle degree of primary roots for WT and *rmd-1* in HP and LP conditions. Error bars mean ± SE, n = three independent biological replicates with 14 roots in each assay were analyzed. In HP, *P* > 0.05 (WT, *rmd-1*) for 1 h, *P* < 0.05 (WT, *rmd-1*) for 2 h, *P* < 0.01 (WT, *rmd-1*) for 4 h and 6 h, *P* > 0.05 (WT, *rmd-1*) for 8 h from Student's *t*-test; In LP, *P* < 0.01 (WT, *rmd-1*) for 1 h, 2 h, 4 h, 6 h and 8 h, from Student's *t*-test; For WT, *P* < 0.05 (HP, LP) for 1 h, *P* < 0.05 (HP, LP) for 2 h, *P* < 0.01 (HP, LP) for 4 h, 6 h and 8 h from Student's *t*-test; For *rmd-1, P* > 0.05 (HP, LP) for 1 h, 2 h, 4 h, 6 h and 8 h from Student's *t*-test. **c**, *RMD AT1* and *RMD AT2* showed less response to HP and LP in gravitropism. Error bars mean ± SE, n = three independent biological replicates with 17 roots analyzed in each assay, two asterisks mean significant difference (*P* < 0.01 from Student's *t*-test). In HP, *P* < 0.01 (WT, *RMD AT1*), *P* < 0.01 (WT, *RMD AT2*), *P* > 0.05 (*RMD AT1*, *RMD AT2*) form Student's *t*-test. In LP, *P* < 0.01 (WT, *RMD AT1*), *P* < 0.01 (WT, *RMD AT2*), *P* > 0.05 (*RMD AT1*, *RMD AT2*) form Student's *t*-test.

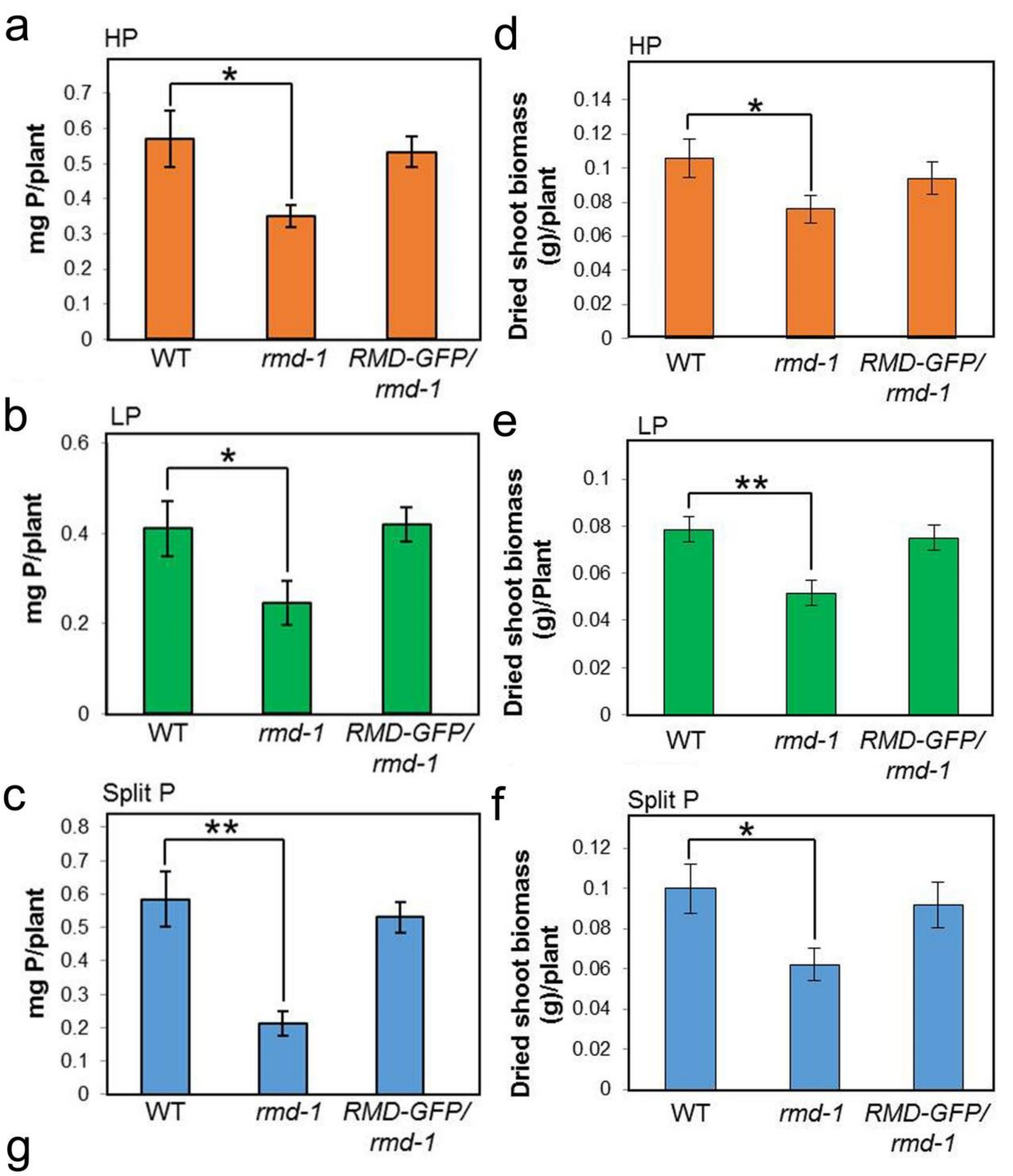

g

Conditions	Biomass reduction in *rmd1* (%)	Reduction in P content in *rmd1* (%)
Split P	37.89	63.75
Low P	40.46	40.13
High P	28.37	38.57

Supplementary Figure 7. *rmd-1* showed reduced total phosphate content and shoot biomass accumulation. (a-c) Total phosphate contents of WT, *rmd-1* and *RMD-GFP/rmd-1* in HP, LP and split phosphate conditions. Error bars are ± SE with 4 independent replicates. Student's *t*-test: *$P < 0.05$; **$P < 0.01$. **(d-f)** Total dry shoot

biomass of WT, *rmd-1* and *RMD-GFP/ rmd-1* lines in HP, LP and split phosphate conditions. Error bars are ± SE of at 6 independent replicates. * and ** indicate $P < 0.05$ and 0.01, respectively. **(g)** Reduced biomass and phosphate content in *rmd-1* as compared to WT. Percentage reduction values were derived from means of WT and *rmd-1* plotted in figure (**a-f**).

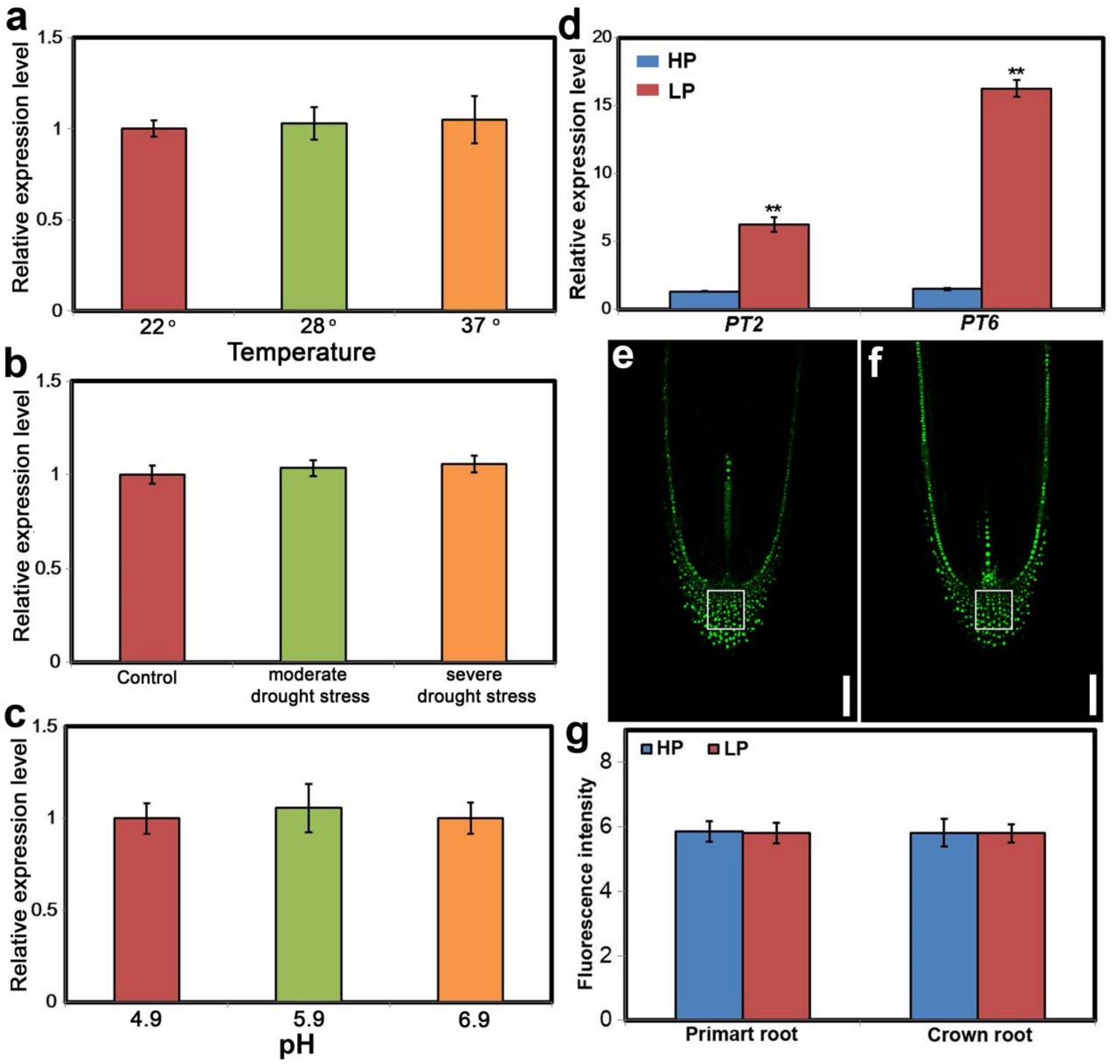

Supplementary Figure 8. The expression level of *RMD* under different conditions. **(a)** RT-qPCR measurement of *RMD* transcript abundance in both primary and crown roots grown under PH 4.9, PH 5.9 and PH 6.9 conditions. Error bars mean ± SE, n = three independent assays. (**b)** RT-qPCR measurement of *RMD* transcript abundance in both primary and crown roots grown under 22 degree, 28 degree and 37 degree centrigrade. Error bars mean ± SE, n = three independent assays. **(c)** RT-qPCR measurement of *RMD* transcript abundance in both primary and crown roots grown under normal condition, moderate drought stress and severe drought stress. Error bars mean ± SE, n = three independent assays. (**d)** RT-qPCR measurement of *PT2* and *PT6*

transcript abundance in both primary and crown roots grown under LP versus HP conditions. Error bars mean ± SE, n = three independent assays. **(e)** The representative image of *DR5::3XVENUS-N7* in WT under HP condition for crown roots, the boxed region was used for analysis. Bar, 100 μm. **(f)** The representative image of *DR5::3XVENUS-N7* in WT under LP condition for crown roots. Bar, 100 μm, the boxed region was used for analysis. **(g)** Fluorescence intensity analysis of *DR5::3XVENUS-N7* in columella cells for primary roots and crown roots under HP and LP. Error bars mean ± SD with 12 roots were analyzed.

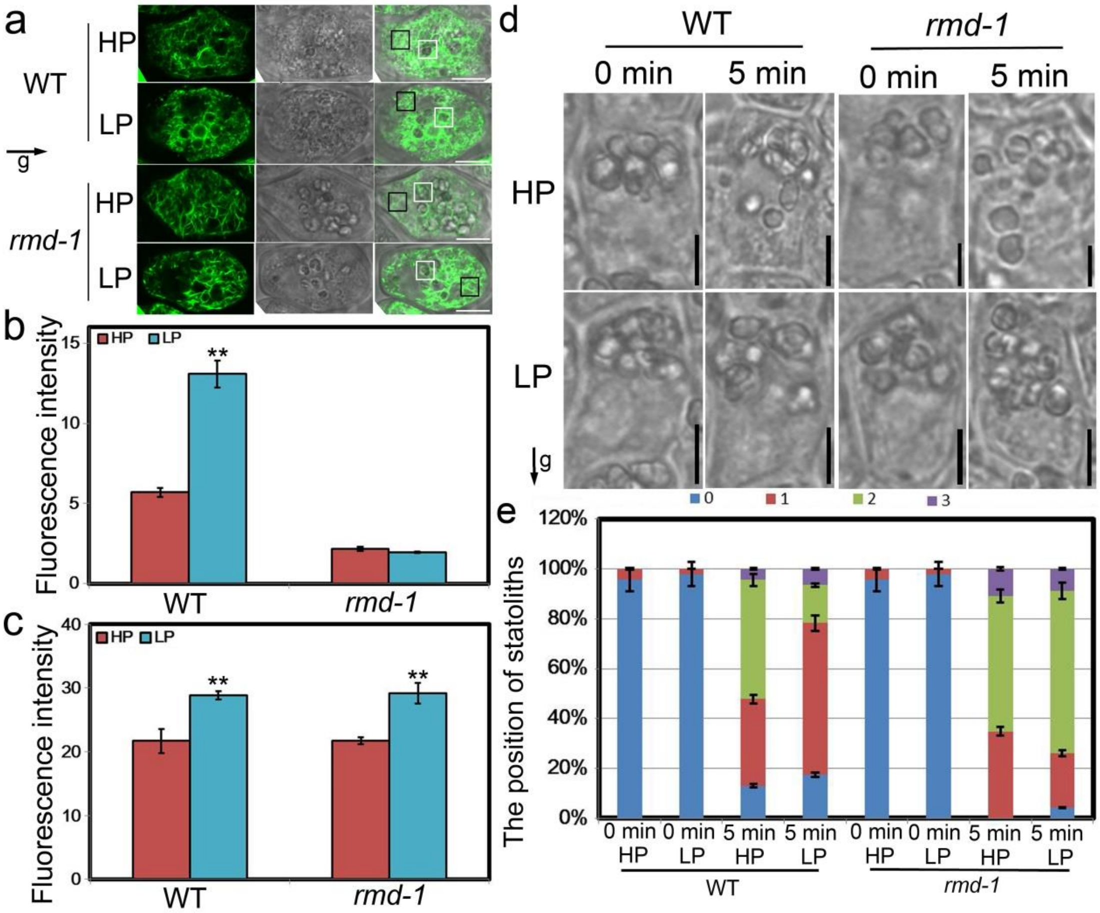

Supplementary Figure 9. The sedimentation of crown roots statoliths is reduced under LP in an RMD-dependent manner. (a) The representative images of crown roots AFs staining of WT and *rmd-1* in HP and LP. Bars, 5 μm. (**b)** Fluorescence intensity of the AFs around statoliths in WT and *rmd-1* in HP and LP. Error bars are mean ±SE, n = 3 independent biological replicates with 11 cells from different roots analyzed in each assay, two asterisks mean significant difference (P < 0.01 from Student's *t*-test). In HP, P < 0.01 (WT, *rmd-1*) form Student's *t*-test. In LP, P < 0.01 (WT, *rmd-1*) form Student's *t*-test. (**c)** Fluorescence intensity of the AFs of cytoplasm in WT and *rmd-1* crown roots in HP and LP. Error bars are mean ±SE, n = 3 independent biological replicates with 13 cells from different roots analyzed in each assay, two asterisks mean significant difference (P < 0.01 from Student's *t*-test). In HP,

$P > 0.05$ (WT, *rmd-1*) form Student's *t*-test. In LP, $P > 0.05$ (WT, *rmd-1*) form Student's *t*-test. **(d)** The representative images of crown roots columella cells of WT and *rmd-1* during gravitropism. Bars, 5 µm. **e**, The quantified position of statoliths during gravitropism. Error bars mean ± SE, n = 3 independent biological replicates with 19 cells from different roots analyzed in each assays.

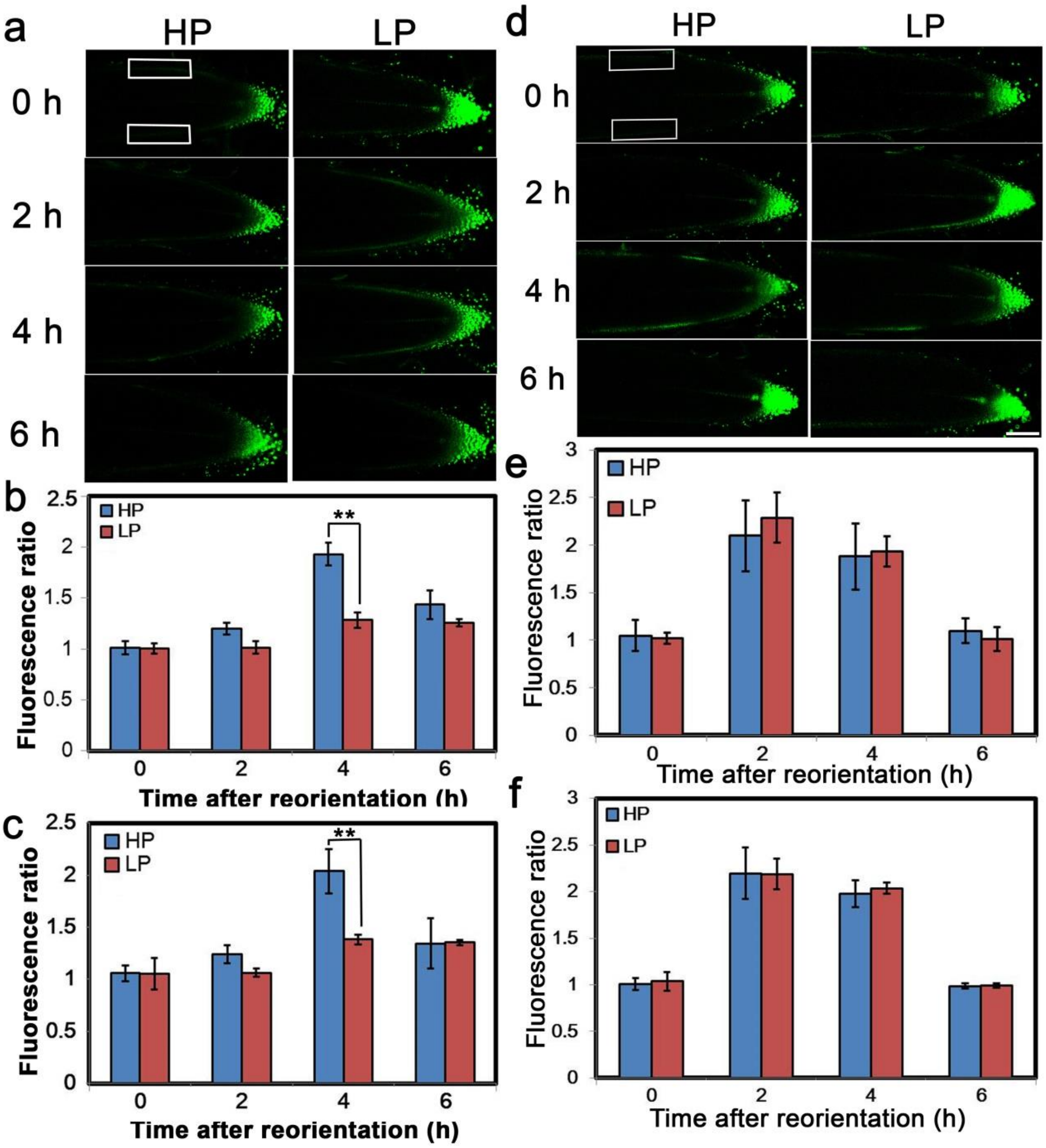

Supplementary Figure 10. Lateral auxin gradient formation in primary roots and crown roots is not altered under HP versus LP conditions in *rmd-2*. (a) Fluorescence distribution of *DR5::3XVENUS-N7* after gravistimulation in crown roots of WT in HP and LP. The boxed regions were analyzed in this assay. Bar, 200 µm. **(b)** Kinetics of the fluorescence intensity ratio of basal side to upper side after gravitropism in primary roots. Error bars are ± SD (n = 15), two asterisks are significant difference ($P < 0.01$ from Student's *t*-test). **(c)** Kinetics of the fluorescence

intensity ratio of basal side versus upper side after gravitropism in crown roots. Error bars are ± SD (n = 18), two asterisks are significant difference (*P* < 0.01 from Student's *t*-test). **(d)** Fluorescence distribution of *DR5::3XVENUS-N7* after gravistimulation in crown roots of *rmd-2* in HP and LP. The boxed regions were analyzed in this assay. Bar, 200 μm. (**e**) Kinetics of the fluorescence intensity ratio of basal side versus upper side after gravitropism in primary roots. Error bars are ± SD (n = 16). (**f**) Kinetics of the fluorescence intensity ratio of basal side to upper side after gravitropism in crown roots. Error bars are ± SD (n = 17).

Supplementary Table 1.

Primers used in this work

Primer name	Sequence (5’ -> 3’)
ZP1	GCTGTACAAGAGATCTATGGCGCTCTTCCGCAAATTCTTC
ZP2	TGCTCACCATACTAGTACCTACATCTTTTCCTCGTCTGC
ZP202	GAGCCTCTGTTCGTCAAGTA
ZP203	ACTCGATGGTCCATTAAACC
ZP190	TAATAGGTAGAGGGCGTGGG
ZP191	CTGACAAATCAAACTCCGAAAC
ZP44	TTATGGAGTTGGGTTCGAACCCCGCAGCAGTATGAAGGAT
ZP45	TAGTTGGAATGGGTTCGAACTGCCCATAACGCTGGAGAT
ZP46	TTATGGAGTTGGGTTCGAACCAGGGATTACTTGAGCGGCA
ZP47	TAGTTGGAATGGGTTCGAAAAACCTCGGAACAACGTGGA
ZP174	GATGTTTCGCTTGGTGGTCG
ZP175	CAAGATGGATTGCACGCAGG
ZP178	CTCGGAGGGCGAAGAATCTC
ZP179	CAATGACCGCTGTTATGCGG
ZP204	GCTGACCACACCTAGCTTTGG
ZP205	AGGGAACCTTAGGCAGCATGT
ZP196	GACGAGACCGCCCAAGAAG
ZP197	TTTTCAGTCACTCACGTCGAGAC
ZP198	GGATCCTTCGGGTTCCTGTA
ZP199	GCGAGCAGGAAGAGCGAG

Supplementary Table 2.

Protocols for HP and LP treatment

1/4 MS	concentration	1x/g	100x/g
NH_4NO_3	5.1537 mM	0.412502148	41.2502148
$H_3B_3O_6$	25.0687 μM	0.001549997721	0.154999772
$CaCl_2$-$2H_2O$	564.8891 μM	0.083049995482	8.304999548
$CoCl_2$-$6H_2O$	29.2715 nM	0.00000696368985	0.000696369
$CuSO_4$-$5H_2O$	25.03 nM	0.000006249991	0.000624999
Na_2 EDTA	25.0242 μM	0.009315008208	0.931500821
$FeSO_4$	25 μM	0.00695	0.695
$MgSO_4$-$7H_2O$	183.2806 μM	0.045175002288	4.517500229
$MnSO_4$-$4H_2O$	18.9411 μM	0.004225001766	0.422500177
H_2MoO_4	385.9216 nM	0.00006250000312	0.00625
KI	1.6566 μM	0.0002749956	0.02749956
KNO_3	4.6983 mM	0.47499813	47.499813
$ZnSO_4$	13.3152 μM	0.002150005344	0.215000534

Solution 2	concentrations	1x/g	100x/g
KH_2PO_4	31.2293 mM	0.042499954	4.249995437

Solution 3	concentrations	1x/g	100x/g
KCl	31.2293 mM	0.023281443	2.328144315

1 liter HP (312 μM) consists of 10 mL 1/4 MS and 10 mL solution 2, PH=5.9. 1 liter 1/2 HP consists of 10 mL 1/4 MS, 5 mL solution 2 and 5 mL solution 3, PH=5.9. 1 liter 1/4 HP consists of 10 mL 1/4 MS, 2.5 mL solution 2 and 7.5 mL solution 3, PH=5.9. 1 liter LP (3.12μM) consists of 10 mL 1/4 MS, 100 μL solution 2 and 10 mL solution 3, PH=5.9.
