# Supplementary material for: Rice actin binding protein RMD controls crown root angle in response to external phosphate
Source: Nat Commun. 2018 Jun 11;9:2346. doi: 10.1038/s41467-018-04710-x (PMC5995806; doi:10.1038/s41467-018-04710-x)
Supplement: Supplementary file 2 — Description of Additional Supplementary Files [file 41467_2018_4710_MOESM2_ESM.pdf]

Legend for Supplementary Movies

Supplementary Movie1

Primary roots of *rmd-1* showed enhanced gravitropism. Compared to wild type, primary roots of *rmd-1* display 90° curvature after 6-h gravitropism. Every second equals to 20 minutes.

Supplementary Movie 2

Crown roots of *rmd-1* showed enhanced gravitropism. Compared to wild type, crowns roots of *rmd-1* exhibited 90° curvature after 6-h gravitropism. Every second equals to 20 minutes.
